# Mizoribine Ameliorates Renal Injury and Hypertension along with the Attenuation of Renal Caspase-1 Expression in Aldosterone-Salt-Treated Rats

**DOI:** 10.1371/journal.pone.0093513

**Published:** 2014-04-02

**Authors:** Toshiki Doi, Shigehiro Doi, Ayumu Nakashima, Toshinori Ueno, Yukio Yokoyama, Nobuoki Kohno, Takao Masaki

**Affiliations:** 1 Department of Nephrology, Hiroshima University Hospital, Hiroshima, Japan; 2 Department of Molecular and Internal Medicine, Graduate School of Biomedical Sciences, Hiroshima University, Hiroshima, Japan; University of Geneva, Switzerland

## Abstract

Aldosterone-salt treatment induces not only hypertension but also extensive inflammation that contributes to fibrosis in the rat kidney. However, the mechanism underlying aldosterone-salt-induced renal inflammation remains unclear. Pyroptosis has recently been identified as a new type of cell death that is accompanied by the activation of inflammatory cytokines. We hypothesized that aldosterone-salt treatment could induce inflammation through pyroptosis and that mizoribine, an effective immunosuppressant, would ameliorate the renal inflammation that would otherwise cause renal fibrosis. Ten days after recovery from left uninephrectomy, rats were given drinking water with 1% sodium chloride. The animals were divided into three groups (n = 7 per group): (1) vehicle infusion group, (2) aldosterone infusion group, or (3) aldosterone infusion plus oral mizoribine group. Aldosterone-salt treatment increased the expression of the nucleotide-binding oligomerization domain, leucine-rich repeat and pyrin domain containing 3 and caspase-1, and also increased the number of terminal deoxynucleotidyl transferase dUTP nick end labeling-positive cells. However, the oral administration of mizoribine attenuated these alterations. Furthermore, mizoribine inhibited hypertension and renal fibrosis, and also attenuated the aldosterone-induced expression of serum/glucocorticoid-regulated kinase and α epithelial sodium channel. These results suggest that caspase-1 activation plays an important role in the development of inflammation induced by aldosterone-salt treatment and that it functions as an anti-inflammatory strategy that protects against renal injury and hypertension.

## Introduction

Aldosterone secreted from the adrenal cortex increases the reabsorption of sodium and water by activating epithelial sodium channels (ENaCs) in the collecting ducts of the kidneys [1]. At present, it is widely recognized that the mineralocorticoid receptor (MR) is expressed in the cells of many tissues, and excess aldosterone can directly cause tissue damage through MR activation. In clinical studies, MR antagonists not only suppressed albumin excretion in hypertensive and diabetic patients [2]–[4], but also reduced the risk of both morbidity and mortality in patients with heart failure [5], [6]. Furthermore, MR antagonists showed a renoprotective effect in several experimental models of kidney disease [7]–[12]. Therefore, excess MR activation is believed to cause the progression of renal injury.

Heptinstall et al. developed a salt-sensitive rat model, which was induced by mineralocorticoid infusion [13]. This model showed pathologic findings of massive interstitial inflammation and fibrosis as well as physiologic features of severe hypertension and urinary protein excretion. Previous studies have described that inflammation plays an important role in the pathogenesis of hypertension, and that its inhibition leads to a reduction of blood pressure in several models [14]. In addition, renal inflammation is reported to be associated with the development and progression of renal fibrosis [15]. These findings indicate that aldosterone-induced inflammation could serve as a therapeutic target for resolving salt-sensitive hypertension and renal fibrosis.

Pyroptosis was recently considered to be a type of caspase-1-dependent programmed cell death that causes rapid plasma membrane rupture and the release of inflammatory cytokines such as interleukin (IL)-1β and IL-18 [16]. Although pyroptosis plays a physiological role in that it serves as a protective host response against infectious disease, pyroptosis-induced inflammation can be detrimental [17]. In fact, several studies have described that pyroptosis is involved in the pathogenesis of several diseases that are characterized by cell death and inflammation, including myocardial and cerebral infarction [18], [19], inflammatory bowel disease [20], and endotoxic shock [21].

Mizoribine is a clinically available immunosuppressant with minimal toxicity. After uptake by lymphocytes, mizoribine is metabolized to mizoribine-5′-phosphate by adenosine kinase [22]. The metabolite inhibits inosine monophosphate dehydrogenase and guanine monophosphate synthetase, which are rate-controlling enzymes in the de novo biosynthesis of purines.

In this article, we provide data showing that caspase-1 activation is involved in cell death and inflammation in aldosterone-salt-treated rats. We also indicate that mizoribine, an effective immunosuppressant, alleviates renal inflammation and pyroptosis in vivo. Furthermore, we show that mizoribine suppresses urinary protein excretion, hypertension, and renal fibrosis.

## Methods

### Ethics Statement

The Institutional Animal Care and Use Committee of Hiroshima University (Hiroshima, Japan) approved all of the experimental protocols (Permit Number: A10-52), and the experiments were performed in accordance with the National Institutes of Health Guidelines on the Use of Laboratory Animals. All efforts were made to minimize animal suffering.

### Animals

Six-week-old male Sprague-Dawley rats (220–250 g), obtained from Charles River Laboratories (Yokohama, Japan), were subjected to left nephrectomy under sedation with sodium pentobarbital anesthesia. After 10 days of recovery from surgery, the rats were given drinking water with 1% sodium chloride. The animals were divided into three groups (n = 7 each): the vehicle group receiving vehicle alone, the ALD group receiving an infusion of aldosterone (Sigma, St. Louis, MO, USA) at 0.75 μg/h, and the ALD + MZR group receiving an aldosterone infusion plus mizoribine (Asahi Kasei, Tokyo, Japan) at 3 mg⋅kg^−1^⋅day^−1^ by gavage feeding. An osmotic minipump (Alza, Palo Alto, CA, USA) containing either vehicle alone or aldosterone was inserted subcutaneously under sedation with sodium pentobarbital anesthesia.

The systolic blood pressure (SBP) was measured fortnightly by the tail cuff method (BP-98A; Softron, Tokyo, Japan). We collected 24-h urine samples by using metabolic cages at 14 days after the addition of 1% sodium chloride to the drinking water and 2 days before the end of the experiment. At the end of week 6, blood samples and kidney tissues were collected under sedation with sodium pentobarbital anesthesia.

### Histological examination

Sections of formalin-fixed, paraffin-embedded tissues were stained with periodic acid-Schiff (PAS) reagent. For evaluation of glomerulosclerosis, glomeruli were graded on a scale of 0 to 4: 0, normal; 1, involvement of 1%–25% of the glomerular tufts; 2, involvement of 26%–50% of the glomerular tufts; 3, involvement of 51%–75% of the glomerular tufts; and 4, involvement of 75%–100% of the glomerular tufts. In each animal, 50 glomeruli were graded and the mean value was calculated as the glomerulosclerosis score. For the evaluation of tubulointerstitial injury, the extent of damage (tubular dilatation, protein casts, interstitial fibrosis, and inflammatory cell infiltration) to the entire renal cortex was graded on a scale of 0 to 4: 0, normal; 1, involvement of 1%–25% of the cortex; 2, involvement of 26%–50% of the cortex; 3, involvement of 51%–75% of the cortex; and 4, involvement of 75%–100% of the cortex. This was defined as the tubulointerstitial injury score.

### Immunohistochemistry

The following primary antibodies were used: rabbit polyclonal anti-human CD3 antibody (Dako, Glostrup, Denmark), mouse monoclonal anti-CD68 antibody (Serotec, Oxford, UK), rabbit polyclonal anti-collagen type I antibody (Abcam, Cambridge, UK), and mouse monoclonal anti-α-smooth muscle actin (α-SMA) antibody (Sigma). T lymphocytes (CD3-positive cells) and collagen type I were identified with the EnVision System (Dako). Macrophages (CD68-positive cells) and myofibroblasts (α-SMA-positive cells) were identified with the peroxidase anti-peroxidase method. Details of the methods used have been published previously [23].

DNA strand breaks were detected in paraffin sections by using the DeadEnd Colorimetric TUNEL System (Promega, Madison, WI, USA) according to the manufacturer's instructions.

CD3-positive cells, CD68-positive cells, and terminal deoxynucleotidyl transferase dUTP nick end labeling (TUNEL)-positive cells were counted in eight selected fields of the cortex (×100) that were captured using a digital camera. The area of α-SMA and collagen type I staining was assessed with Lumina Vision 2.20 (Mitani, Osaka, Japan) in four selected fields of the cortex (×40) and four fields of the corticomedullary junction (×40) that were also captured using a digital camera.

### Western blot

Sample collection and immunoblotting were performed as previously described [24]. The following primary antibodies were used: rabbit polyclonal anti-caspase-1 antibody (Millipore, Temecula, CA, USA); rabbit polyclonal anti-nucleotide-binding oligomerization domain, leucine-rich repeat and pyrin domain-containing 3 (NLRP3) antibody (Novus Biologicals, Littleton, CO, USA); mouse monoclonal anti-α-tubulin antibody (Sigma); rabbit polyclonal anti-αENaC antibody (Santa Cruz Biotechnology, Santa Cruz, CA, USA); rabbit polyclonal anti-serum-glucocorticoid regulated kinase (SGK) antibody (Cell Signaling Technology, Beverly, MA, USA); and rabbit polyclonal anti-with-no-lysine 4 (WNK4) antibody (Millipore). The intensity of each band was determined using ImageJ software (version 1.44p; National Institutes of Health).

### RNA extraction and quantitative real-time reverse transcriptase polymerase chain reaction (RT-PCR)

Extraction of total RNA, synthesis of cDNA, PCR, and data analysis were performed as described previously [23]. Gene-specific oligonucleotide primers and probes for interferon (IFN)-γ (assay ID: Rn00594078_m1), tumor necrosis factor (TNF)-α (assay ID: Rn01525859_g1), monocyte chemotactic protein (MCP)-1 (assay ID: Rn01456716_g1), IL-1β (assay ID: Rn00580432_m1), and glyceraldehyde-3-phosphate dehydrogenase (GAPDH) (assay ID: Rn99999916_s1) as an internal control were obtained using TaqMan Gene Expression Assays from Applied Biosystems (Foster City, CA, USA).

### Statistical analysis

Results are expressed as the mean ± SEM for each group of seven rats. Statistical analysis was performed by analysis of variance, followed by Tukey's post hoc test. Data differences were deemed significant at *P*<0.05.

## Results

### Mizoribine suppresses aldosterone-induced sodium retention and proteinuria

We assessed the effect of mizoribine on the physiological characteristics of the aldosterone-salt-treated rats. Urine volume, sodium intake, sodium excretion, and sodium balance at 14 days after the addition of 1% sodium chloride to the drinking water are presented in Table 1. Aldosterone significantly increased sodium retention, whereas mizoribine attenuated this alteration. Moreover, sodium balance was statistically similar in the three groups at the end of the 6-week experiment (data not shown).

**Table 1:** Biological markers at 14 days after the addition of 1% sodium chloride to the drinking water.

	Vehicle group	ALD group	ALD + MZR group
Urine volume (mL)	47±3.9	74±14	44±4.0
Sodium intake (mmol)	10.7±1.1	17.0±3.1	11.3±1.0
Sodium excretion (mmol)	10.8±1.0	14.7±2.7	10.9±0.7
Sodium balance (mmol)	−0.10±0.53	2.33±0.57*	0.40±0.49†

ALD, aldosterone; ALD + MZR, aldosterone + mizoribine

Values are presented as mean ± SEM.

*P<0.05 vs. vehicle group.

†*P*<0.05 vs. ALD group.

The body weight, kidney weight/body weight ratio, urine volume, serum creatinine, creatinine clearance, and urinary protein excretion of each group at the end of the 6-week experiment are shown in Table 2. Aldosterone-salt-treated rats showed marked renal hypertrophy and severe proteinuria. In contrast, renal hypertrophy and proteinuria were prevented by using mizoribine. A high level of serum creatinine was observed in the aldosterone-salt treatment group compared with the other groups, whereas the creatinine clearance did not differ among these groups.

**Table 2:** Data for the three groups at the end of the 6-week experiment.

	Vehicle group	ALD group	ALD + MZR group
Body weight (g)	494±14	414±12*	407±13*
Kidney weight/body weight (%)	0.45±0.02	1.15±0.12*	0.73±0.04* †
Serum creatinine (mg/dL)	0.39±0.02	0.56±0.05*	0.32±0.02†
Urine volume (mL)	46.8±5.9	81.0±12.3*	44.6±5.3†
Creatinine clearance (mL⋅min^−1^⋅kg^−1^)	6.61±0.25	5.29±0.53	6.33±0.43
Urinary protein excretion (mg/day)	0.76±0.09	19.13±3.11*	1.30±0.29†

ALD, aldosterone; ALD + MZR, aldosterone + mizoribine.

Values are presented as mean ± SEM.

*P<0.05 vs. vehicle group.

†*P*<0.05 vs. ALD group.

### Mizoribine suppresses renal inflammation and pyroptosis caused by aldosterone-salt treatment *in vivo*

**Figure 1:**
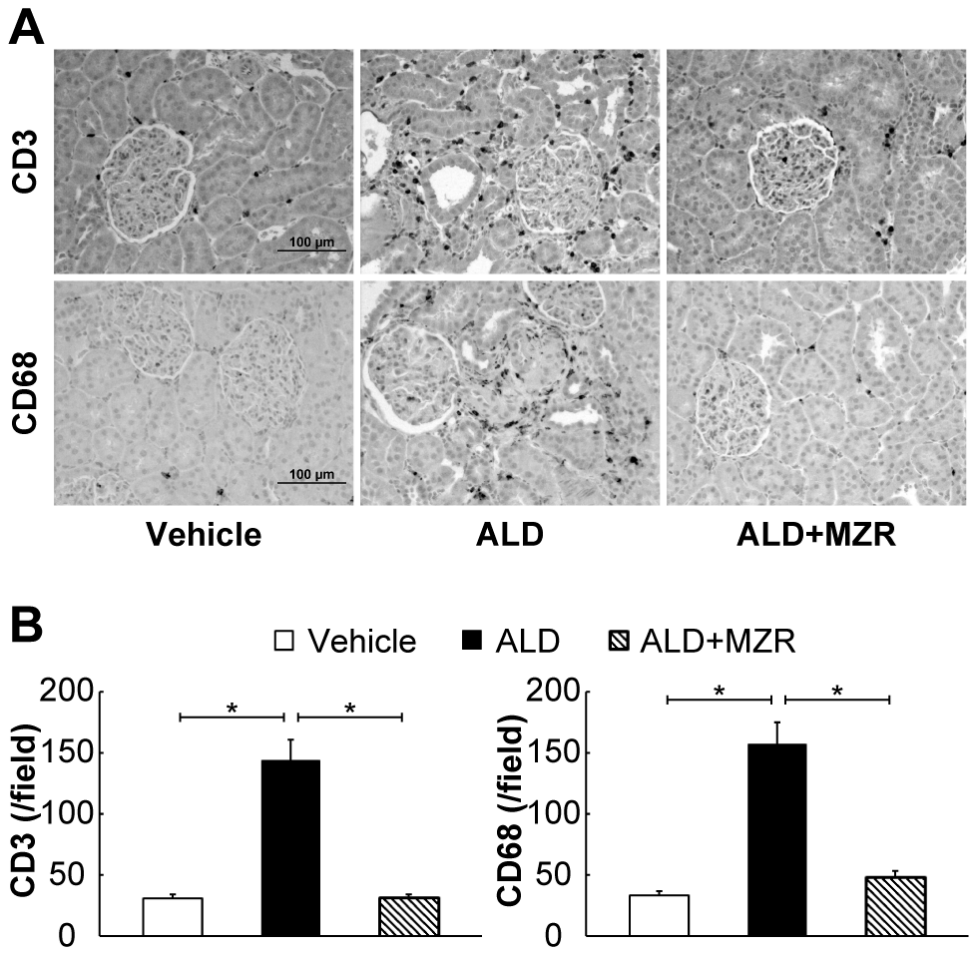
Mizoribine suppresses aldosterone-induced renal inflammation. Aldosterone (ALD) induced considerable T lymphocyte and macrophage infiltration in the rat kidneys, whereas the infiltration was suppressed by mizoribine (MZR). (A) Representative photomicrographs of renal T lymphocyte infiltration (CD3 staining) and macrophage infiltration (CD68 staining). (B) Graphs show the quantification of CD3-positive and CD68-positive cells. Values are presented as mean ± SEM. *P<0.05.

To evaluate the infiltration of inflammatory cells into the rat kidney, we examined the expression of CD3 and CD68, which are markers of T lymphocytes and macrophages, respectively. Aldosterone-salt-treated rats showed considerable T lymphocyte and macrophage infiltration in the kidneys (Figure 1). In contrast, T lymphocyte and macrophage infiltration was suppressed by mizoribine.

**Figure 2:**
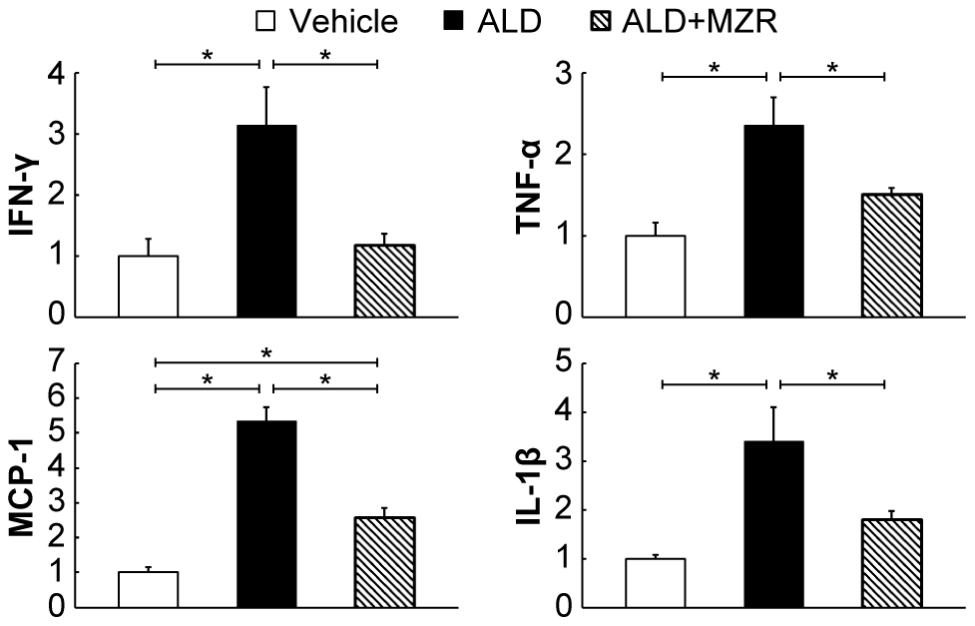
Mizoribine suppresses the renal expression of genes for various aldosterone-induced pro-inflammatory cytokines. Aldosterone (ALD) increased the renal cortical expression of genes for interferon (IFN)-γ, tumor necrosis factor (TNF)-α, monocyte chemotactic protein (MCP)-1, and interleukin (IL)-1β, against that of glyceraldehyde-3-phosphate dehydrogenase (GAPDH). In contrast, the expression of these genes was suppressed by mizoribine (MZR). Values are presented as mean ± SEM. *P<0.05.

Next, we examined the expression of genes for various pro-inflammatory cytokines by quantitative RT-PCR. The expression of IFN-γ, TNF-α, MCP-1, and IL-1β was increased by aldosterone-salt treatment. In contrast, the expression of these genes was suppressed by mizoribine (Figure 2).

**Figure 3:**
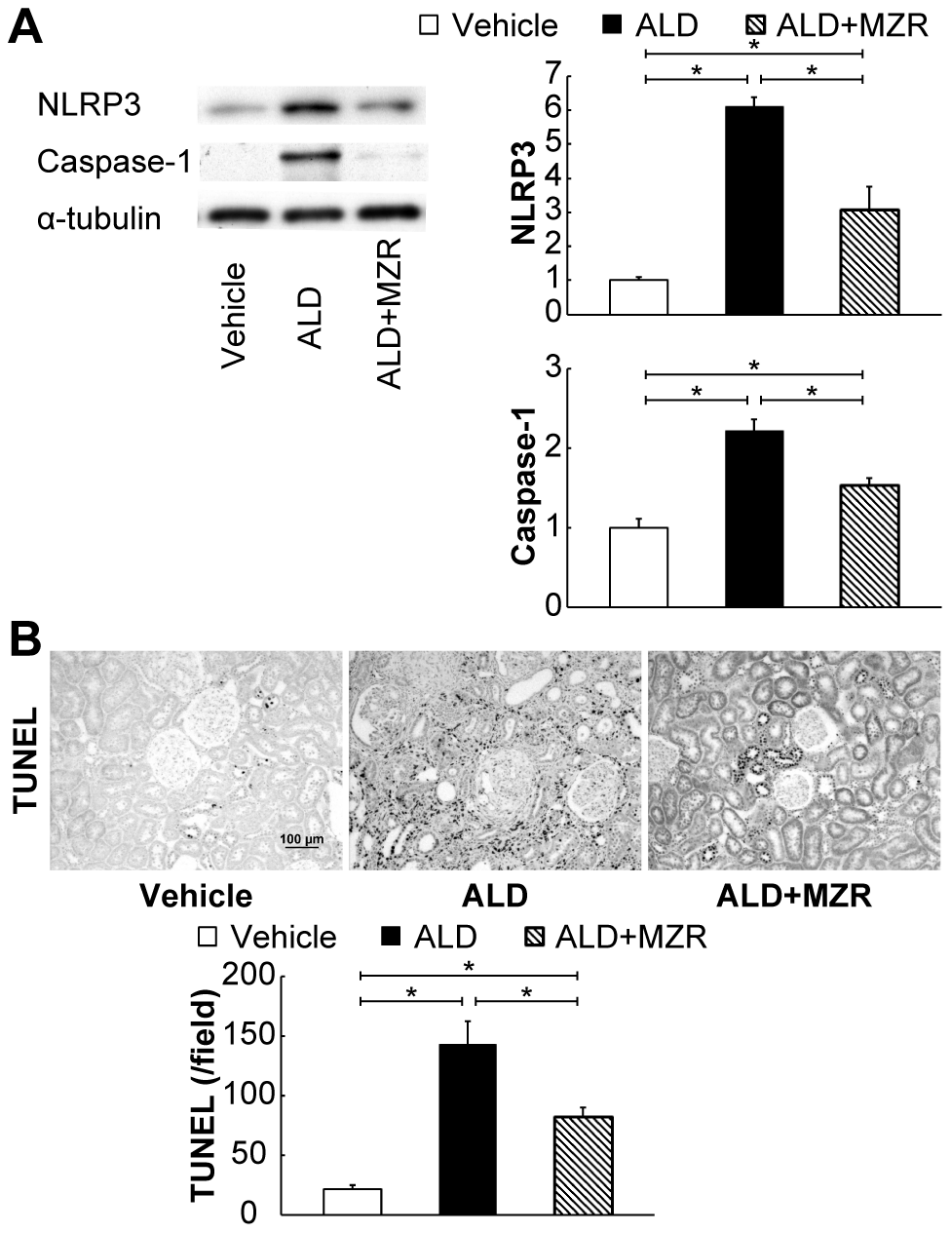
Mizoribine suppresses aldosterone-induced renal pyroptosis. Aldosterone (ALD) enhanced the renal expression of the nucleotide-binding oligomerization domain, leucine-rich repeat and pyrin domain containing (NLRP) 3 and caspase-1, and increased the number of TUNEL-positive cells—the hallmark of pyroptosis—whereas the increase in these factors was significantly suppressed by mizoribine (MZR). (A) Western blot analysis for NLRP3; caspase-1; and α-tubulin was performed. The total cell lysate was prepared from the rat kidney cortex. Graphs show the expression level quantified by densitometry and normalized with α-tubulin. (B) Representative photomicrographs of TUNEL staining of kidney sections. The graph shows the quantification of TUNEL-positive cells. Values are presented as mean ± SEM. *P<0.05.

Recently, it was revealed that a multiprotein complex called the inflammasome induces caspase-1 activation and IL-1β production [16]. The inflammasome consists of NLRP, the apoptosis-associated speck-like protein (ASC), and procaspase-1. The processing of procaspase-1 by the inflammasome triggers a specific pro-inflammatory cell death called “pyroptosis.” Aldosterone-salt treatment enhanced the expression of NLRP3 and caspase-1, and increased the number of TUNEL-positive cells—the hallmark of pyroptosis—whereas mizoribine significantly suppressed the increase in these factors (Figure 3).

### Mizoribine ameliorates hypertension and attenuates the increase of αENaC expression

**Figure 4:**
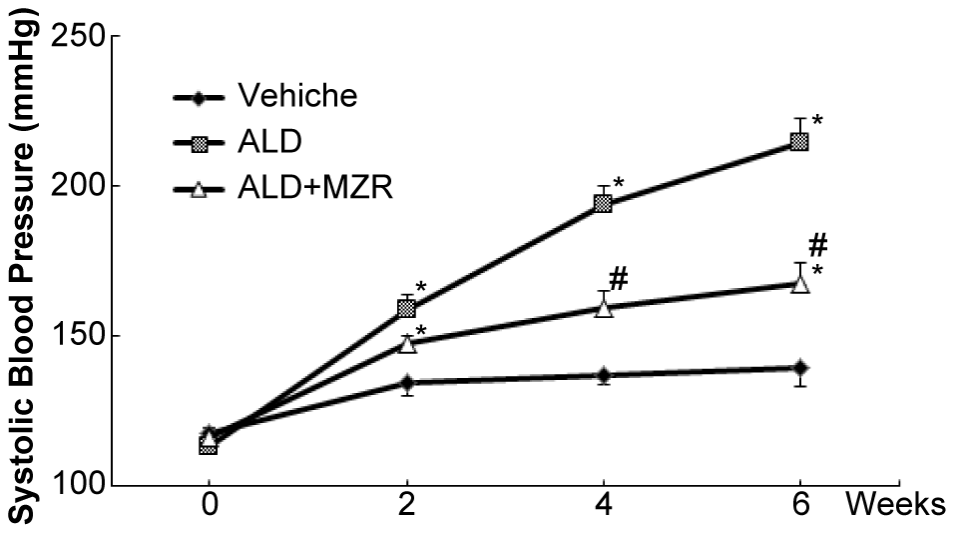
Mizoribine ameliorates aldosterone-induced hypertension. Following infusion of aldosterone (ALD), hypertension developed over time. In contrast, mizoribine (MZR) attenuated the degree of hypertension caused by ALD. Values are presented as mean ± SEM. *P<0.05 vs. vehicle group; #*P*<0.05 vs. ALD group.

We measured the SBP to assess the effect of mizoribine on aldosterone-salt-induced hypertension in rats. At the beginning of the study, the SBP of the three groups was similar (Figure 4). In the aldosterone-salt treatment group, hypertension developed over time. Mizoribine attenuated the degree of hypertension caused by aldosterone-salt treatment.

Aldosterone leads to hypertension and increases the reabsorption of sodium by activating ENaC. Of the several pathways through which aldosterone activates ENaC, SGK is known to play a central role in ENaC expression. In addition, WNK4 is known as a downstream effector of SGK and is known to inhibit ENaC activity.

**Figure 5:**
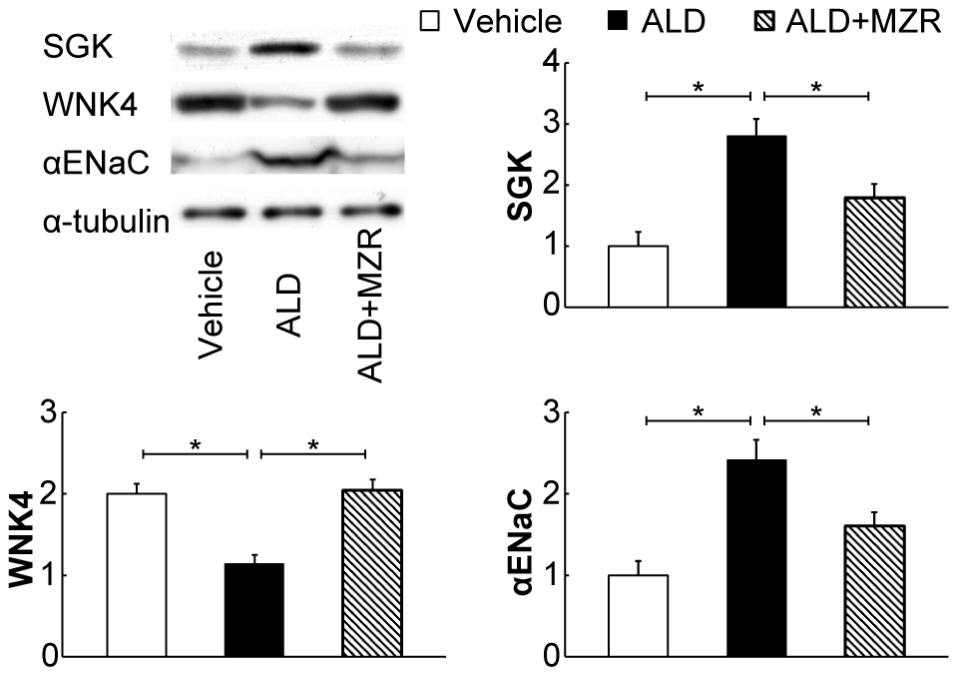
Mizoribine attenuates aldosterone-induced upregulation of serum/glucocorticoid-regulated kinase and α epithelial sodium channels. Western blot analysis for serum/glucocorticoid-regulated kinase (SGK), with-no-lysine 4 (WNK4), α epithelial sodium channel (αENaC), and α-tubulin was performed. The total cell lysate was prepared from the renal cortex of rats. Aldosterone (ALD) increased the expression of SGK and αENaC, and decreased the expression of WNK4, whereas mizoribine (MZR) attenuated these alterations. Graphs show the expression level quantified by densitometry and normalized with α-tubulin. Values are presented as mean ± SEM. *P<0.05.

Aldosterone-salt treatment increased the expression of SGK and αENaC, and decreased the expression of WNK4, whereas mizoribine administration attenuated these alterations (Figure 5).

### Mizoribine attenuates histological renal damage and renal fibrosis

**Figure 6:**
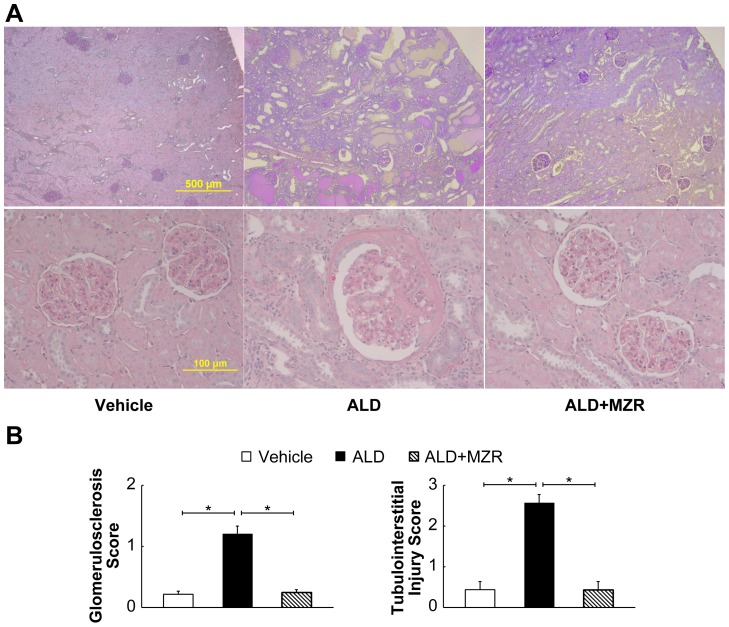
Mizoribine attenuates histological aldosterone-induced renal damage. Aldosterone (ALD) induced severe glomerular and tubulointerstitial injury, whereas mizoribine (MZR) markedly attenuated the injury. (A) Representative photomicrographs of glomeruli and tubulointerstitial areas with periodic acid-Schiff staining. (B) Graphs show the quantification of glomeruli and tubulointerstitial injury. Values are presented as mean ± SEM. *P<0.05.

PAS staining was used to assess the changes in renal tissue and to analyze the renal damage. Severe glomerular and tubulointerstitial injury was detected in aldosterone-salt-treated rats (Figure 6). Glomerular injury included the presence of glomerular sclerosis, mesangiolysis, hemorrhage, expansion of glomerular tufts, and glomerular hypertrophy, whereas tubulointerstitial injury included tubular dilatation, protein casts, interstitial fibrosis, and inflammatory cell infiltration. Mizoribine markedly attenuated glomerular or tubulointerstitial injury.

**Figure 7:**
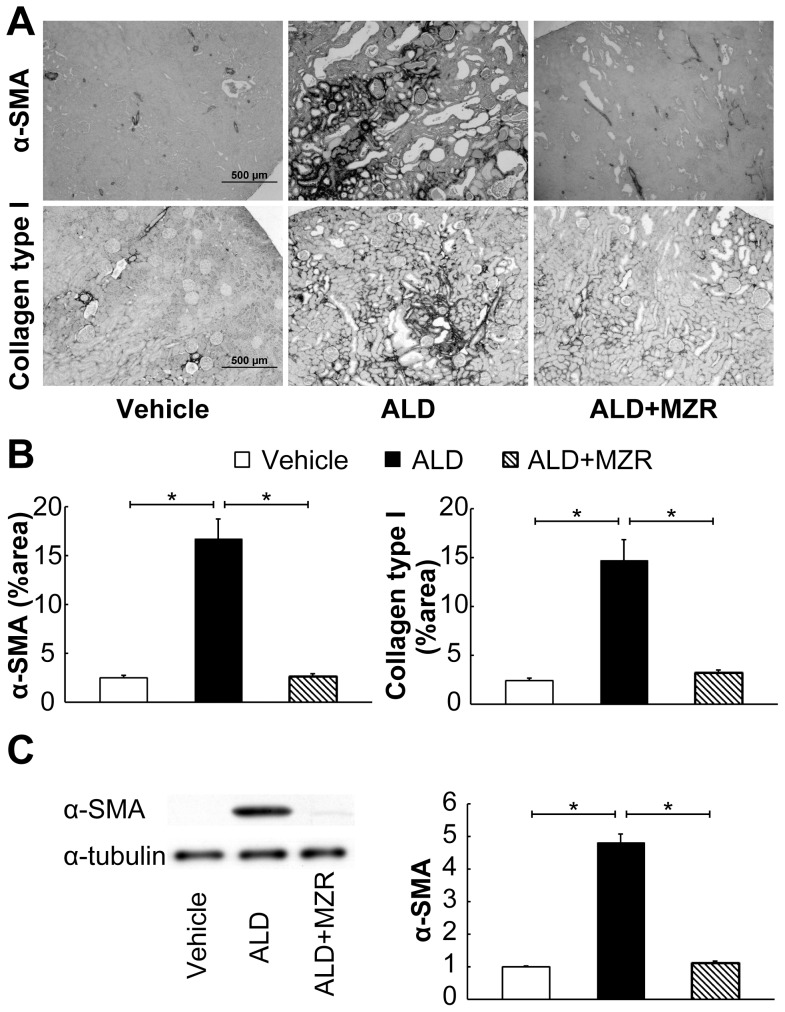
Mizoribine attenuates aldosterone-induced renal fibrosis. Aldosterone (ALD) increased the renal expression of collagen type I and α-smooth muscle actin (α-SMA), whereas mizoribine (MZR) significantly suppressed the expression. (A) Representative photomicrographs of collagen type I- and α-SMA-positive areas of the kidney section. (B) Graphs show the collagen type I- and α-SMA-positive percent areas. (C) Western blot analysis for α-SMA and α-tubulin was performed. The total cell lysate was prepared from the renal cortex of rats. Graphs show the expression level quantified by densitometry and normalized with α-tubulin. Values are presented as mean ± SEM. *P<0.05.

To evaluate renal fibrosis, we examined the expression of α-SMA and collagen type I, which are markers of myofibroblasts and extracellular matrix proteins, respectively (Figure 7). In the vehicle group, α-SMA expression was detected in the smooth muscle cells of renal arterioles, but not in the interstitium. Although the aldosterone-salt-treated group showed an increase in interstitial α-SMA expression, this expression was suppressed by mizoribine treatment, to a level comparable to that in the vehicle group. As in the case of α-SMA, aldosterone-salt treatment increased the expression of collagen type I, and mizoribine inhibited this expression.

## Discussion

The present study demonstrated that immunosuppressant therapy with mizoribine alleviates renal inflammation and cell death accompanied by caspase-1 activation in aldosterone-salt-treated rats. Mizoribine also shows beneficial effects on hypertension, urinary protein excretion, and renal fibrosis. Therefore, mizoribine may be a therapeutic option for salt-sensitive hypertension and renal fibrosis.

Renal fibrosis is thought to be linked to inflammation in aldosterone-salt treatment models [25]. The data of this study suggest that mizoribine inhibits the accumulation of inflammatory cells and prevents the upregulation of IFN-γ, TNF-α, and MCP-1 mRNA induced by the aldosterone infusion. IFN-γ secreted by effector T lymphocytes has a potent influence on macrophage activation [26], and the activated macrophages produce pro-inflammatory cytokines such as TNF-α and MCP-1 [27]. A number of studies have reported that the inhibition of inflammatory cytokines such as TNF-α and MCP-1 ameliorates renal injury in several experimental models [28]–[32]. These findings support the concept that TNF-α and MCP-1 secreted by activated macrophages in the kidneys cause tissue damage.

The expression of SGK, caspase-1, NLRP3, and IL-1β mRNA, as well as the number of TUNEL-positive cells were increased by the aldosterone infusion in the kidneys of the salt-loaded rats. Inflammasomes are multiprotein complexes that serve as a platform for caspase-1 activation, which results in TUNEL-positive cell death and release of inflammatory cytokines. The priming step in the formation of the NLRP3 inflammasome is provided by endogenous molecules that activate nuclear factor κB (NF-κB) to induce pro-IL-1β and NLRP3. Previous reports have clarified that aldosterone stimulates NF-κB by activating SGK [33], [34]. Moreover, the actual activation mechanism is poorly understood even though the involvement of the K^+^ efflux, elevated reactive oxygen species levels, and lysosomal rupture has been established. However, it likely involved the integration of a number of signals that are indicative of cellular damage or stress. Recently, mitochondrial dysfunction has been considered as one of the factors that activate the NLRP3 inflammasome, and aldosterone is known to induce renal mitochondrial dysfunction [35], [36]. Taken together, the mechanism by which aldosterone induces pyroptosis likely involves SGK activation, which then promotes the expression of pro-IL-1β and NLRP3, as well as mitochondrial dysfunction, which then induces caspase-1 activation through the formation of the NLRP3 inflammasome.

Aldosterone is well-known as an activator of ENaC, which is involved in rate-limiting sodium reabsorption in the distal nephron. We demonstrated that mizoribine suppresses renal αENaC expression in aldosterone-salt-treated rats. We also observed that aldosterone induced sodium retention, which then disappeared at the end of the 6-week experiment. This phenomenon, termed “aldosterone escape,” is attributable to several events, such as increased renal perfusion pressure, increased sodium delivery to the distal nephron sites of mineralocorticoid action, and volume expansion-induced elevation of plasma natriuretic hormone level [37]. In the present study, blood pressure in aldosterone-salt-treated rats showed a significant increase during the observation periods, which may play an important role in sodium excretion.

SGK is known to prevent Nedd4-2-induced ENaC ubiquitination, resulting in increased ENaC expression [38]. Recently, WNKs, a new member of the serine/threonine kinase family, were found to be involved in the regulation of sodium, potassium, and blood pressure. ENaC is considered to be activated by WNK1 but inhibited by WNK4. In the present study, the renal expression of SGK and αENaC was increased by aldosterone infusion, whereas WNK4 expression was decreased. Moreover, mizoribine administration attenuated the SGK and αENaC upregulation and the WNK4 downregulation. Therefore, mizoribine suppresses the aldosterone-induced reabsorption of sodium by attenuating the degree of change in the expression of these factors.

In the present study, mizoribine was found to ameliorate aldosterone-induced hypertension and also inhibit inflammation. Inflammatory cells have been found to accumulate in the kidneys in several experimental models of hypertension, including in aldosterone-infused rats, and the process of inflammation seems to have an important role in the development of hypertension [14]. Guzik et al. reported that the induction of hypertension through treatment with angiotensin II or deoxycorticosterone acetate is suppressed in rag-1^−/−^ mice, which lack T and B lymphocytes [39]. They also showed that effector T lymphocytes have an important role in these models and that etanercept, a TNF-α inhibitor, can prevent hypertension induced by angiotensin II treatment. These reports support our findings that inhibition of the inflammatory response improves aldosterone-induced hypertension.

We found that mizoribine suppresses aldosterone-induced fibrosis in the kidneys. Renal fibrosis is known as a final common pathway of various chronic kidney diseases, whereas renal inflammation is known to contribute to fibrosis. Zhang et al. reported that aldosterone induces epithelial-mesenchymal transdifferentiation (EMT) [40]. Cheng et al. also showed that unilateral urinary obstruction-induced EMT was suppressed in SGK-knockout mice [41]. These reports suggest that aldosterone-induced inflammation and SGK play critical roles in the development of renal fibrosis. In addition to inflammation, hypertension is recognized as one of the most significant factors of renal injury [42], indicating that a reduction of blood pressure during mizoribine treatment participates, at least partly, in its beneficial effect on renal fibrosis.

Studies have demonstrated that mizoribine suppresses inflammation, thereby protecting against various experimental insults [43]–[46]. Our resulting data show that mizoribine suppresses not only inflammation but also fibrosis and hypertension. In the clinical setting, mizoribine is administered as an immunosuppressant and no statement that it may have adverse effects on blood pressure is included in the drug information, suggesting that mizoribine may reduce renal injury and blood pressure via an immunosuppressive effect. Interestingly, the present study shows that mizoribine attenuates aldosterone-induced SGK and αENaC upregulation in vivo. However we could not clarify the possibility that mizoribine directly influences the expression of SGK and αENaC. Thus, further studies are required to assess the manner in which mizoribine attenuates aldosterone-induced SGK and αENaC upregulation.

In the present study, we demonstrated that aldosterone induces inflammation with caspase-1 activation in response to salt loading. Because the activation of caspase-1 leads to pyroptosis and release of activated cytokines, we believe that the formation of the NLRP3 inflammasome may contribute to renal inflammation in aldosterone-salt-treated rats. In addition, mizoribine alleviates not only renal inflammation but also hypertension and renal fibrosis. In conclusion, caspase-1 activation may play an important role in the development of inflammation and pyroptotic cell death, which may result in hypertension and renal fibrosis in aldosterone-salt-treated rats. These results suggest that immunosuppressants could be a useful therapy for salt-sensitive hypertension and renal injury.
